# Exploring room temperature spin transport under band gap opening in bilayer graphene

**DOI:** 10.1038/s41598-023-36800-2

**Published:** 2023-06-26

**Authors:** Christopher R. Anderson, Noel Natera-Cordero, Victor H. Guarochico-Moreira, Irina V. Grigorieva, Ivan J. Vera-Marun

**Affiliations:** 1 Department of Physics and Astronomy, University of Manchester, Manchester, M13 9PL UK; 2 Consejo Nacional de Ciencia y Tecnología (CONACyT), Mexico City, Mexico; 3 Facultad de Ciencias Naturales y Matemáticas, Escuela Superior Politécnica del Litoral, ESPOL, Campus Gustavo Galindo, Km. 30.5 Vía Perimetral, P.O. Box 09-01-5863, 090902 Guayaquil, Ecuador; 4 Center of Nanotechnology Research and Development (CIDNA), Escuela Superior Politécnica del Litoral, ESPOL, Campus Gustavo Galindo Km 30.5 Vía Perimetral, Guayaquil, Ecuador; 5 National Graphene Institute, University of Manchester, Manchester, M13 9PL UK

**Keywords:** Spintronics, Electronic properties and devices

## Abstract

We study the room-temperature electrical control of charge and spin transport in high-quality bilayer graphene, fully encapsulated with hBN and contacted via 1D spin injectors. We show that spin transport in this device architecture is measurable at room temperature and its spin transport parameters can be modulated by opening of a band gap via a perpendicular displacement field. The modulation of the spin current is dominated by the control of the spin relaxation time with displacement field, demonstrating the basic operation of a spin-based field-effect transistor.

## Concept and novelty

**Figure 1:**
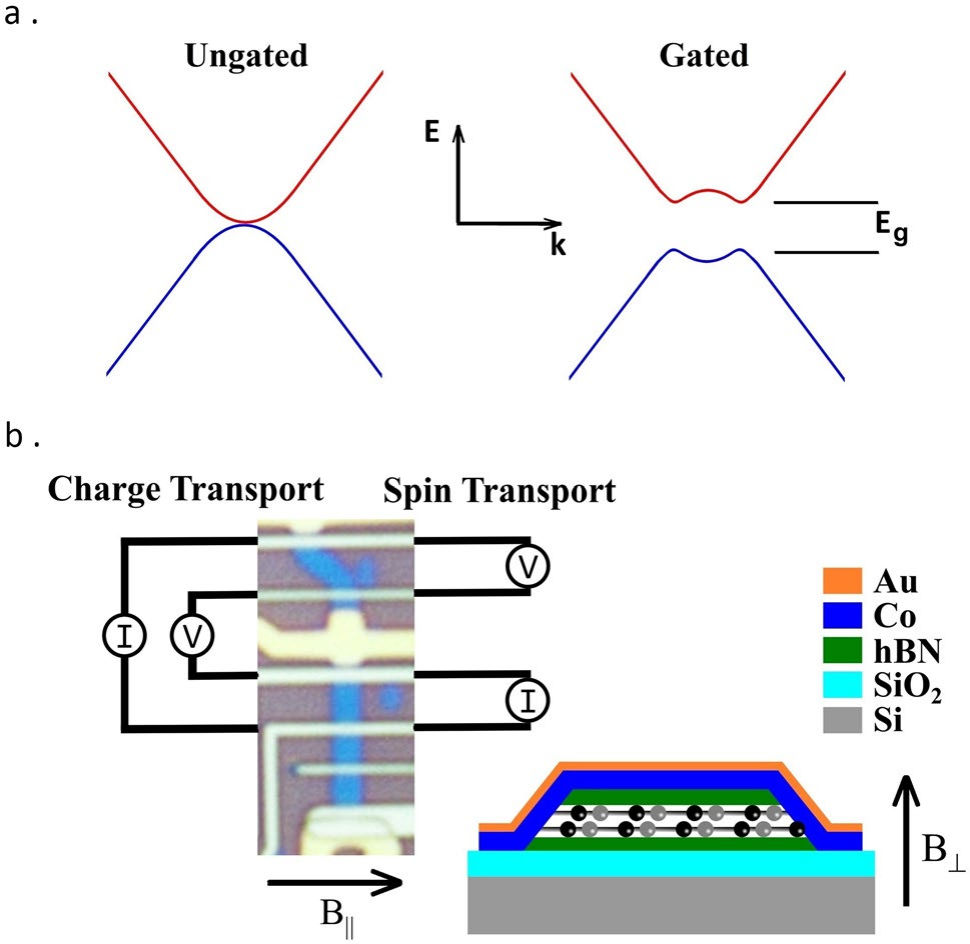
Bilayer graphene transport channel and device. (**a**) Band structure of pristine BLG without (left) and with (right) an applied perpendicular electric displacement field. (**b**) Optical micrograph of our $\sim$1 µm wide BLG graphene transport channels (blue) with contacts and top gate (in the measurement region). The charge (spin) transport current injection, *I*, and local (non-local) potential difference, *V*, measurement configuration. Inset: Schematic of the device heterostructure showing the Co/Au 1D edge contacts, with the graphene represented by the balls and sticks.

The state variable in digital devices has been realised, to date, by measurement of charge. Spintronics offers a new paradigm, whereby the quantum spin states of one or more electrons could be used as an alternative; improving performance by quick and efficient manipulation of that spin state^1^. Improvements in transport and control of spin are necessary for spin logic to be fully realised. Graphene offers desirable properties including theoretically large, although experimentally small spin ‘quality’ parameters^2,3^. Bilayer graphene (BLG) presents the opportunity to electrically control its band gap^4,5^, $E_g$, (see Fig. 1a) and, as a result, the spin transport parameters in the channel. Graphene based spin-based field effect transistors have been previously attempted: Materials with large and electrostatically tunable spin-orbit coupling (SOC) have been brought into contact with a graphene channel, enabling spins to be absorbed^6,7^; this precludes the fabrication of a fully encapsulated homogeneous transport channel. A combination of injector current and back gate voltage can be tuned to create a spin transistor-like action by reversing the polarity of the contact spin polarisation^8,9^, however, the latter varied between contacts and consequently so did the spin signal, making this approach not scalable. High-quality graphene devices with reproducible contact spin polarisation can overcome this limitation^10^. Alternatively, the electrostatic control of the graphene channel’s band gap^11–13^ offers a small, but promising effect, which has the potential to be scalable whilst maintaining a simple architecture, with excellent transport properties.

Spin transport has previously been observed in single layer (SLG) and BLG devices which have transparent (invasive) contacts (exhibiting the spin resistance mismatch problem^14,15^) and a SiO$_{2}$ substrate^16–18^ which brakes graphene’s inversion symmetry causing Rashba fields, increased SOC and spin relaxation. Alternatively, devices have been fabricated with an hBN substrate but are not fully encapsulated^12^, leading to a heterogeneous channel consisting of sections with and without encapsulation that suffer from environmental degradation. Quantum tunnelling contacts have been shown to solve the resistance mismatch problem^11,12,17,19,20^ improving the measured spin transport parameters. However, such tunneling contacts are difficult to fabricate due to troublesome selection of atomically thin hBN^21^ or inconsistency of the growth of an atomically thin barrier^17^.

The effect which we have observed, where we propose that spin anisotropy is induced in the BLG by a small but tuneable band gap, has previously been seen but at high carrier densities and low temperatures^16^. In contrast, here we experimentally study a bilayer device at room temperature (RT) and show how, close to the Dirac point, the transport parameters are effected by an applied perpendicular displacement field which induces the band gap to open (Fig. 1a). In this regime, however, it is typically difficult to measure the device’s spin transport parameters, as a consequence of the low signal to noise ratio and an induced band gap commensurate with the thermal activation energy. Conversely, at the Dirac point the effects of the band gap opening on spin transport are more readily observed, with the spin splitting being more prominent at the band edge. Consequently, the quality of the homogeneous channel is of paramount importance, which allows the observation of a modulation of the spin relaxation time, $\tau _s$, which has not previously been shown close to the Dirac point and at RT. Here this is made possible by the fabrication of a BLG transport channel which is fully encapsulated, where the effect of the contacts and the resistance mismatch problem are minimised by the use of nanoscale 1D edge contacts^9,10^.

## Results

### Device architecture and fabrication

The full encapsulation of the BLG with hBN leaves only the sandwiched graphene’s edges exposed; on these edges 1D contacts are formed (Fig. 1b). The device was designed to enable electrostatic gating of the whole device using the Si substrate as a back gate and of a region between two contacts using a microfabricated top gate. The design facilitates the fabrication of multiple fully encapsulated regions with top gate electrodes. The 1D edge magnetic contacts are fabricated from gold-capped cobalt, whilst the electrostatic top gates are gold, with a thin chromium adhesion layer.

### Charge transport measurements

**Figure 2:**
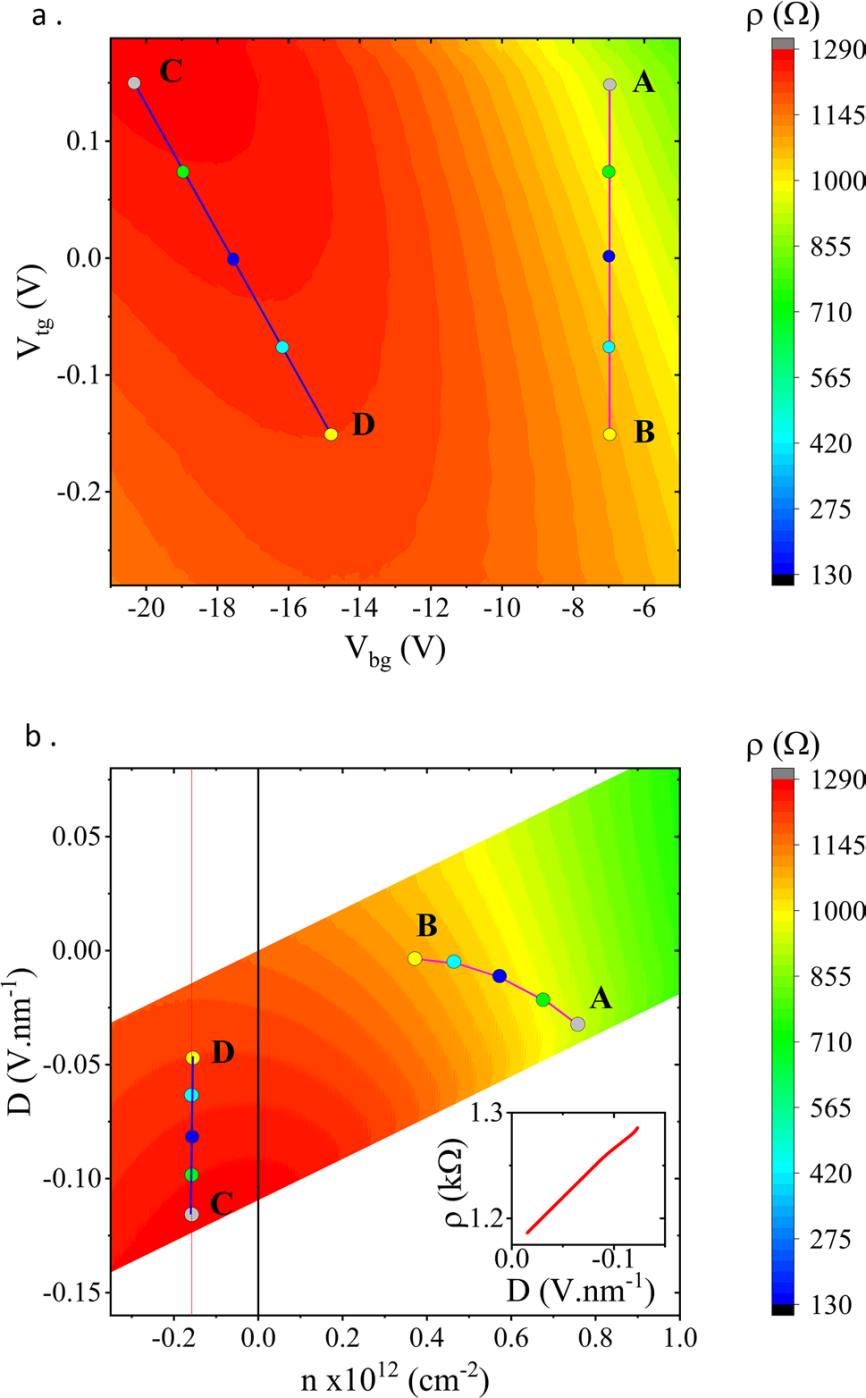
(**a**) A 2D charge transport measurement map at RT showing the effect on the sheet resistance, $\rho$, of applying a back gate voltage, $V_{bg}$, and top gate voltage, $V_{tg}$. (**b**) The same charge transport measurements transformed into a map as a function of carrier density, *n*, and electric displacement field, *D*. In both maps the symbols are shown where the spin transport measurements were made. b**(inset)** Sheet resistance close and parallel to the Dirac ridge ($n \approx 0$).

Our local measurements of the top-gated and hBN-encapsulated BLG, using the configuration shown in Fig. 1b, explored the charge transport by varying the back gate, $V_{bg}$, and top gate, $V_{tg}$, voltages and measuring the BLG resistance, *R*. From the latter the sheet resistance, $\rho$, is calculated and exhibits a modulation as a function of the gate voltages as shown in Fig. 2a. The gate voltages are used to calculate the carrier density, *n*, and electrical displacement field, *D*, which enable re-plotting the sheet resistance in terms of *n* and *D*. Figure 2a,b show this transformed map at RT, along with symbols indicating a selection of (*n*, *D*) pairs where spin transport measurements were performed. The inset of Fig. 2b shows a near linear relationship between the transport channel’s sheet resistance and the applied displacement field, close and parallel to the Dirac ridge (the path of zero carrier density as the displacement field is modulated), exhibiting a monotonic increase in $\rho$ as *D* increases in magnitude.

The device shows a carrier field effect mobility, $\mu$, of up to 40,000 cm$^{2}$/Vs at 20 K and 11,000 cm$^{2}$/Vs at RT; where $\mu = (d\sigma /{dn})/e$ and $\sigma = 1/\rho$ and are evaluated at a moderate carrier density, $|n| \sim 1 \times 10^{12}$ cm$^{-2}$. These values are comparable with those achieved in fully encapsulated BLG, tunnel barrier contacted devices^11,19,20^ and substantially higher than non-encapsulated BLG devices^12,16^. The BLG transport channel does not suffer from any significant contact doping due to the small contact area (a few carbon atoms depth from the edge of the device) and small contact width ($\le 400$ nm)^10^ and minimal bubbles within the heterostructure. We propose that even higher mobilities may be obtained with the fabrication of a wider channel and eradication of any residual bubbles in the channel, potentially improving the spin transport parameters of the device, however, this is dependent on the cause of the scattering^11^. We have confirmed that the top gate voltage has little effect on the resistance of the regions either side of the top gated region.

### Spin transport measurements

**Figure 3:**
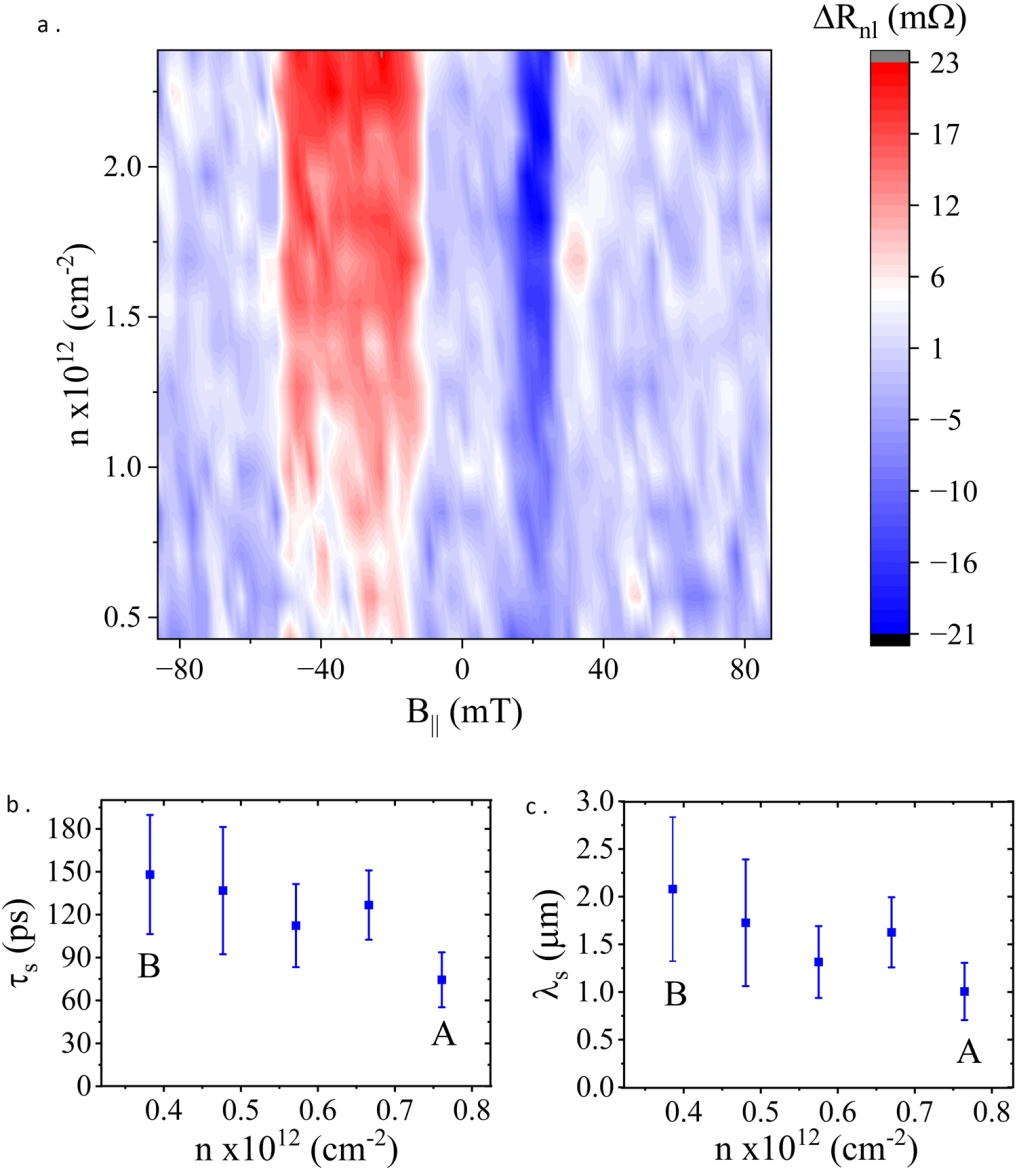
(**a**) 2D map of spin valves at RT as the electron carrier density is increased. The anti-parallel magnetic alignments are clearly visible as the vertical red and darker blue bands. As the CNP is approached the signal becomes increasingly weak. (**b**,**c**). Spin transport parameters ($\tau _s$ and $\lambda _s$, respectively) extracted from spin precession measurements at RT, for the 5 points along the A–B path shown in Fig. 2.

To characterise spin transport we first measured a series of spin valves, using the non-local configuration shown in Fig. 1b, at RT. These are measurements where we sweep an in-plane magnetic field, $\text{B}_\parallel$, applied along the direction of contacts, which reverses the magnetisation of the 1D contacts and enables us to obtain either a parallel or antiparallel magnetic alignment between the injector and detector contacts. The trace and retrace scans of magnetic field are then subtracted from one another to obtain a purely spin-dependent signal. Such measurements were repeated across a carrier density range ($n= 0.45$ to $0.8 \times 10^{12}$ cm$^{-2}$), with the top gate grounded, the results of which are shown in Fig. 3a. The vertical bands of colour seen to the left (red) and right (darker blue) of zero magnetic field strength, $B = 0$ T, are the distinctive spin-valve signals as the magnetic injector/detector configurations change from parallel to anti-parallel. The average magnitude of the spin signal, the difference in non-local resistance between parallel and anti-parallel magnetic configurations, $\Delta R_{nl}$, across this range of $V_{bg}$ is $\sim$10 m$\Omega$. A close examination of the 2D map shows that the spin signal and the signal to noise ratio (SNR) diminish as the back gate approaches the charge neutrality point (CNP, $n = 0$) from the electron carrier regime. This is a characteristic of 1D edge contacts, whose spin polarisation approaches zero near the CNP^9^.

**Figure 4:**
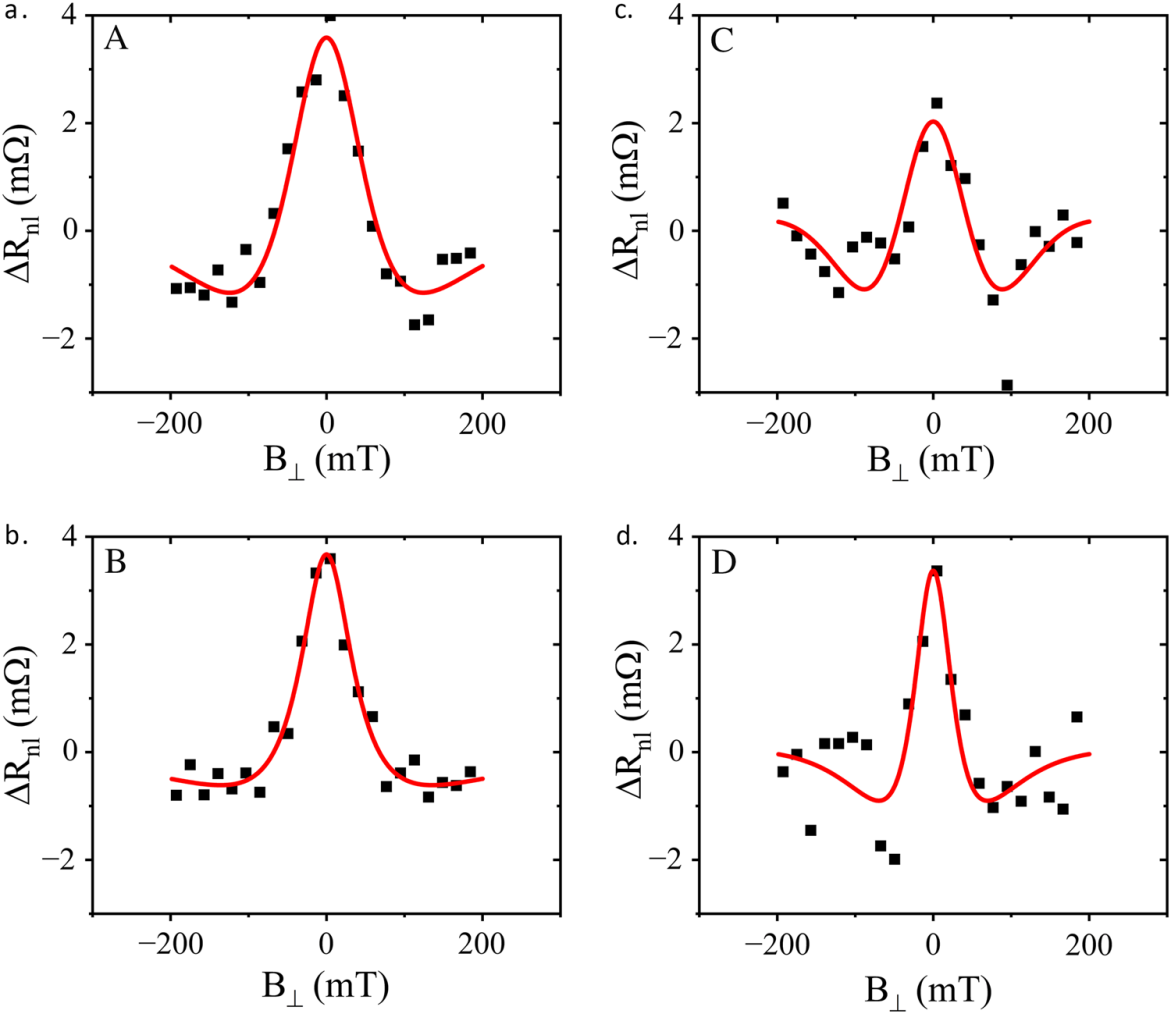
Spin precession measurements at RT. The lineshape of the Hanle curve changes with the change in *n*, shown in A and B, whilst C and D (at $n \approx$ 0) shows the lineshapes when the displacement field changes. The labels correspond to the same (*n*, *D*) pairs labelled in Fig. 2. The Hanle equation fits are shown in red.

**Figure 5:**
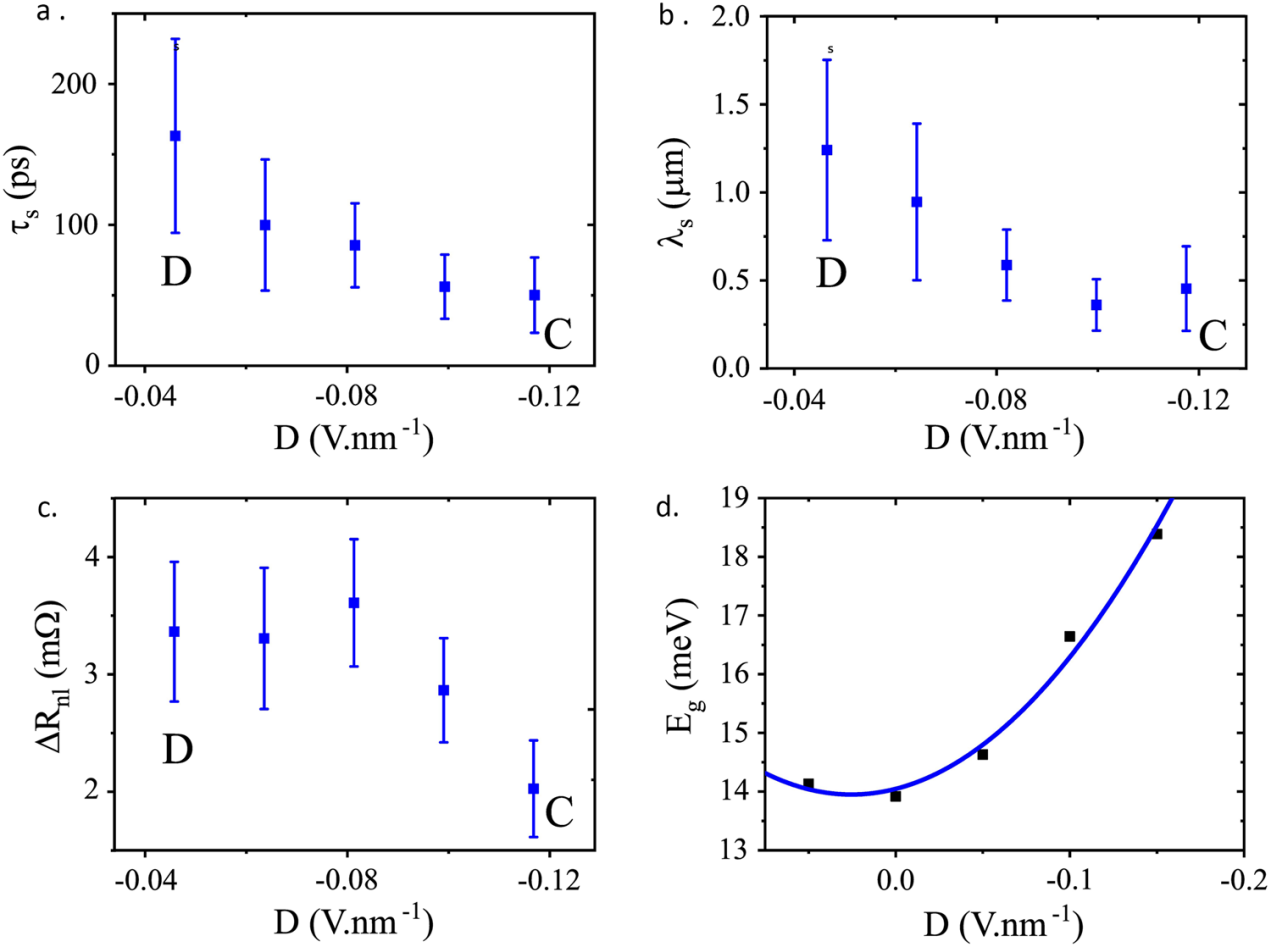
Spin transport measurement and analysis close and parallel to the Dirac ridge at RT. Spin relaxation time (**a**), spin relaxation length (**b**) and spin signal (**c**) for the five points along the C–D path shown in Fig. 2. (**d**) Band gap energy, $E_g$, vs displacement field, *D*, from Arrhenius analysis of charge transport measurements.

The changes in spin signal as a function of carrier density were further studied by performing spin precession measurements. In doing so, we sweep magnetic field strength perpendicular to the plane of the graphene (see Fig. 1b), $\text{B}_\perp$, across a range of ±200 mT, which causes the diffusing electronic spins to experience Larmor precession, and fit the spin signal with the Hanle equation^22^. The spin relaxation time, the spin diffusion coefficient and the corresponding spin relaxation length, are extracted from the Hanle curve fits (examples shown in Fig. 4). Uncertainties in determining these parameters are realised by the error bars in Figs. 3b, c and 5. We use the spin, rather than the charge diffusion coefficient in our analysis, as the measurements are made close to the neutrality point, whilst also opening a band gap, consequently it is inappropriate to use the Einstein relation to extract the charge diffusion coefficient from charge transport measurements. We select five pairs of (*n*, *D*) values for spin transport measurements in the electrons regime, at fixed top gate voltage and $-7$ V$_{\textrm{bg}}$, where carrier density variation dominates over variation in displacement field. The selected five points are shown in Fig. 2(A–B path). The corresponding spin transport measurements and Hanle fits are shown in Fig. 4a,b (A and B) and the extracted spin transport parameters are shown in Fig. 3b,c. Here we note that the spin relaxation time, $\tau _s$, and spin relaxation length, ${\lambda _s}$, decrease with increasing carrier density (at low carrier density) which, although small, is commensurate with that typically observed for BLG spin devices^11^.

Next, we explore spin transport near charge neutrality under the effect of a displacement field. For this purpose we measured spin precession at five different (*n*, *D*) values indicated by the C–D path shown in Fig. 2a,b. Here we fixed a carrier density close to the CNP, $n<2\times 10^{11}$ cm$^{-2}$, and varied *D* from $-0.04$ V nm$^{-1}$ to $-0.12$ V nm$^{-1}$. Examples of the spin precession measurements are shown in Fig. 4 c,d (C and D). A wider peak is seen at the high end of the displacement field range (C) and a narrower peak at the low end of the range (D). These changes in lineshape correspond to a modulation in the spin transport parameters extracted from the Hanle analysis. The spin transport parameters: spin relaxation time, $\tau _s$, spin relaxation length, $\lambda _s$, and spin signal (at *B* = 0), are shown in Fig. 5a–c, respectively. As the magnitude of *D* is increased there is a threefold decrease in $\tau _s$, and a corresponding modulation of $\lambda _s$.

## Discussion

The spin signal, $\Delta R_{nl}$, measured along the Dirac ridge ($n\sim 0$) at RT, shows a decrease with increasing displacement field (Fig. 5c), with a modulation of approximately half.

To understand the role of the applied displacement field, *D*, we measured a series of maps of local resistance versus *n* and *D* from 20 K up to RT. This enables the study of thermal excitation of carriers for a given *D*, with a fixed $n\approx 0$. Arrhenius analysis^16,23,24^ of these measurements yielded the value of the band gap in the encapsulated BLG channel, exhibiting band gap values of up to $\sim 19$ meV as the magnitude of the displacement field increases to 0.2 V nm$^{-1}$ (see Fig. 5d). A finite band gap at zero displacement field applied is noted, along with a non-linear relationship between the magnitude of *D* and $E_g$, which may be caused by the presence of the hBN encapsulation. These results are commensurate with previous work on BLG^4,16^.

Having evidenced the role of the displacement field in opening a band gap in the BLG channel, we proceed to consider how this relates to the changes observed in spin transport at RT. We note that the dependence of $\tau _s$, $\lambda _s$ and $\Delta R_{nl}$ on *D* is correlated with the shape of the relationship between $E_g$ and *D*. At low *D* there is little change in $E_g$; the larger changes in $E_g$ only being seen at higher *D* (Fig. 5d). Therefore, within the transport parameter error bars shown (Fig. 5a–c), we propose that for $|D| \lesssim$ 0.08 V nm$^{-1}$ reveal no effect of the displacement field, however, for $|D|>$ 0.08 V nm$^{-1}$ we begin to see an effect. A displacement field, beyond the range shown, was limited by potential damage to the device.

At RT and close to the Dirac point it is typically difficult to measure the device’s spin transport parameters, as a consequence of the low signal to noise ratio, the resistance mismatch problem^25^ and an induced band gap which is commensurate with the thermal activation energy. However, at the Dirac point the effects of the band gap are more readily observed, with the spin splitting being more prominent at the band edge in BLG^26^.

It should be noted that our measurement configuration is designed to evaluate the difference in energy between the spin up and spin down directly, rather than attempting to measure the spin up and spin down accumulations independently. Consequently, it is sensitive to small net spin accumulations, of the order of $\sim$ µeV. Also, the quality of our homogeneous channel allows us to show a modulation of the spin relaxation time, $\tau _s$, which has not previously been shown. This is made possible by the fabrication of a BLG transport channel which is fully encapsulated, where the effect of the contacts and the resistance mismatch problem are minimised by the use of nanoscale 1D edge contacts^9,10^.

A trivial mechanism by which opening a band gap can modulate spin transport is via the modulation of the charge resistance. An increase of the sheet resistance, $\rho$, in the region where the displacement field is applied, between the injector and detector contacts, would inhibit carrier diffusion and prevent the flow of spin current across this region^11^. We indeed observe a modulation of charge transport at RT, as shown in the inset of Fig. 2b, confirming that the band gap is being electrically opened. Nevertheless, at RT this only accounts for a change of 5% in $\rho$ for the spin transport measurements along the C-D path. The spin diffusion between injector and detector contacts, at a distance $L = 3.0$ µm apart, is described by the following relation^17^,

1 $$\begin{aligned} \Delta R_{nl} \propto \rho \lambda _s e^{-\frac{L}{\lambda _s}}, \end{aligned}$$

from which we would expect, to first order, the spin signal, $\Delta R_{nl}$, to only increase by 5% with increasing displacement field, as it is proportional to $\rho$. On the contrary, we observe that $\Delta R_{nl}$ decreases as the displacement field increases (see Fig. 5c), with an on/off ratio of $\lesssim 2$ from D to C.

A more significant mechanism by which the displacement field can modulate spin transport is via changes in the spin relaxation length, $\lambda _s$. As shown in Eq. (1), the factors containing $\lambda _s$ have the effect of decreasing $\Delta R_{nl}$ as $\lambda _s$ decreases, which is consistent with the observed dependence of $\lambda _s$ on displacement field (see Fig. 5b). Therefore the effect of $\lambda _s$ overwhelms the small effect of $\rho$. Note that ideally, based on a twofold decrease in $\lambda _s$, one would expect a $\Delta R_{nl}$ on/off ratio of up to 200, larger than that observed. We find that this expected modulation in $\Delta R_{nl}$ is, however, muted in our measurements, due to a simultaneous increase in the contact polarisation from 0.5 to 5%, which partially counteracts the effect of $\lambda _s$. The latter is a limitation of our current device design, where the contacts are under the influence of the back gate, and is consistent with a strong dependence of the polarisation of 1D contacts as a function carrier density^9^. Modification of the device design, by introducing a local back gate that does not act on the contacts, would enable a larger on/off ratio due to only the effect of opening a band gap. Given that the charge transport parameters, resistivity and diffusion coefficient, vary only by 5% across our range of displacement field, the variation in the spin relaxation length is given by the dependence $\lambda _s \propto \sqrt{\tau _s}$.

We observe that as the displacement field is increased, the spin relaxation time, $\tau _s$, decreases by up to a threefold, and correspondingly the length, $\lambda _s$ decreases too. This observation is commensurate with the increasing width and decreasing height of the central peak in the spin precession measurements (Fig. 4) as *D* increases. Previous work has demonstrated that in BLG the spin lifetime anisotropy, the ratio of the out-of-plane to the in-plane spin relaxation time, is modulated by a perpendicular displacement field, due to the resulting out-of-plane spin-orbit fields^16^. This previous work showed that the anisotropy increases with increasing displacement field in BLG, as a result of both the increase in out-of-plane spin relaxation time and the decrease in the in-plane spin relaxation time. However, unlike our experiment, this was carried out at high carrier densities and low temperatures.

Given our measurement geometry determines the transport of in-plane spins^17^, such a change in anisotropy due out-of-plane spin-orbit fields in BLG is commensurate with our observations shown in Fig. 5a. Note that this result of a perpendicular displacement field is opposite to that present in SLG, where it results in an effective in-plane Rashba spi-orbit field that causes dephasing of the out-of-plane spins, i.e. the opposite effect on spin anisotropy^27^. On the other hand, in BLG the dominant phenomena is that of the change in band structure due to opening the band gap, which allows spin splitting at the band edges and gives rise to the observed modulation of spin relaxation time with displacement field^16^. The spin transport measurements and analysis described above, presented in Fig. 5b–d, show that it is possible to modulate the spin signal in a BLG spin transistor operating at RT, by virtue of the resulting modulation in $\tau _s$ when opening the band gap.

## Conclusion

We have shown that in a fully hBN encapsulated, 1D edge contacted BLG at RT, it is possible to electrostatically modulate a nonlocal spin current purely by a displacement field. The latter contributes to opening a band gap, with only a small modulation of the charge transport, but a sizeable modulation of the spin relaxation time and corresponding spin signal.

This work, using a van der Waals device architecture which ensures minimally invasive contacts and scalable design of electrostatic gates, paves the way towards further exploration of bilayer graphene as a prototypical spin-FET at RT, which has application in spin logic^28^. SOC effects are now of significant interest in technological areas of spintronics such as for memory devices^29^. Further, investigation of the combination of proximity induced SOC with the tuneable band gap SOC afforded by BLG and/or a drift current^30^ may produce an enhanced on/off ratio and spin current guiding.

## Methods

### Sample fabrication

BLG samples were prepared by mechanical exfoliation of natural graphite^31^, using the so called Scotch tape method and transferred onto a Si/SiO$_{2}$ (290 nm) substrate. BLG candidates were initially found using optical microscopy and the number of layers confirmed with Raman spectroscopy. Similarly hBN candidates were identified, confirming their thickness with a combination of AFM and profilometry. The bottom hBN flake of our devices has a thickness of 12.5 nm and the top flake 5.5 nm. Using the dry transfer technique, a Poly Methyl Methacrilate (PMMA) membrane ($\sim$500 nm thick) lifted the top hBN flake, assisted by a transfer rig equipped with micromanipulators and a hot plate, which improved the membrane stickiness. This top hBN, on the membrane, was used to lift the BLG with van der Waals forces. Finally both flakes were deposited on top of the bottom hBN, already on the Si/SiO$_{2}$ substrate. The remaining PMMA membrane was removed by soaking the sample in acetone and IPA for $\sim$5 minutes each. The stack was annealed in a H_2_/Ar atmosphere for 3 hours at 300 $^{\circ }$ C to remove PMMA residues and contamination. AFM imaging qualitatively determined the cleanest areas of the stack (clean, bubble free) where the graphene spin channel could be patterned. A double PMMA electron beam lithography (EBL) resist layer (solutions of 3% of each of 495 and 950 molecular weight) was spin-coated onto the sample. The channel was patterned with EBL and etched with an O$_{2}$/CHF$_{3}$ plasma, leaving the graphene’s edges exposed, ready to accept 1D edge contacts. Finally, contacts were patterned using EBL and Cr/Au evaporated to form non-magnetic contacts, whilst Co/Au was used to form ferromagnetic contacts with a range of aspect ratios.

### Transport measurement

All spin transport measurements were made in a standard non-local lateral 4-point configuration. Spin precession measurements were fitted with the Hanle equation and spin parameters extracted, the results of which are shown in Fig. 5b–d. The measurements were made by setting the contact magnetic configuration to parallel and anti-parallel using an in-plane magnetic field in a non-local spin valve configuration. In each of the parallel and anti-parallel configurations an out of plane magnetic field was swept and the non-local resistance, $R_{nl}$, recorded. The top and back gate voltages, $V_{tg}$ and $V_{bg}$, were stepped to achieve the required carrier density, *n*, and electric displacement field, *D*, according to Eqs. (2) and (3)^16^,

2 $$\begin{aligned} n&= \epsilon _0 \epsilon _{r_{bg}} \frac{V_{bg} - V_{D_{bg}}}{e t_{bg}} + \epsilon _0 \epsilon _{r_{tg}} \frac{V_{tg} - V_{D_{tg}}}{e t_{tg}} \end{aligned}$$

3 $$\begin{aligned} D&= \epsilon _{r_{bg}} \frac{V_{bg} - V_{D_{bg}}}{2 t_{bg}} + \epsilon _{r_{tg}} \frac{V_{tg} - V_{D_{tg}}}{2 t_{tg}} \end{aligned}$$

where $V_{D}$ is the top gate, *tg*, and back gate, *bg*, voltage required to overcome residual channel doping and achieve charge neutrality. *t* is the thickness of the gate dielectric and $\epsilon _{r}$ is the relative permittivity of the gate dielectric, which can be calculated from the respective gate’s capacitance, *C*, equations,

4 $$\begin{aligned} C_{bg}&= \frac{\epsilon _{r_{bg}}}{t_{bg}} A =\frac{\epsilon _{r_{SiO_2}} \epsilon _{r_{bg hBN}}}{\epsilon _{r_{SiO_2}} t_{bg hBN} + \epsilon _{r_{gb hBN}} t_{SiO_2}} A \end{aligned}$$

5 $$\begin{aligned} C_{tg}&= \frac{\epsilon _{r_{tg}}}{t_{tg}} A =\frac{\epsilon _{r_{tg hBN}}}{t_{tg hBN}} A \end{aligned}$$

where *A* is the area of the gate, ${\epsilon _{r_{SiO_2}}}$ = 3.9 and ${\epsilon _{r_{bg hBN}}}= {\epsilon _{r_{tg hBN}}}$ = 3.2 are the relative permittivities of the SiO$_{2}$ substrate and hBN, respectively. $t_{tg hBN}$ and $t_{bg hBN}$ are the thicknesses of the two hBN flakes, described earlier. The device presented here has a SiO$_{2}$ thickness, ${t_{SiO_2}}$ = 290 nm.

## Data Availability

The datasets used and/or analysed during the current study are available from the corresponding author on reasonable request.
