# Author Correction: Exploring room temperature spin transport under band gap opening in bilayer graphene

**DOI:** 10.1038/s41598-023-42109-x

**Published:** 2023-09-11

**Authors:** Christopher R. Anderson, Noel Natera-Cordero, Victor H. Guarochico-Moreira, Irina V. Grigorieva, Ivan J. Vera-Marun

**Affiliations:** 1 Department of Physics and Astronomy, University of Manchester, Manchester, M13 9PL UK; 2 Consejo Nacional de Ciencia y Tecnología (CONACyT), Mexico City, Mexico; 3 Facultad de Ciencias Naturales y Matemáticas, Escuela Superior Politécnica del Litoral, ESPOL, Campus Gustavo Galindo, Km. 30.5 Vía Perimetral, P.O. Box 09-01-5863, 090902 Guayaquil, Ecuador; 4 Center of Nanotechnology Research and Development (CIDNA), Escuela Superior Politécnica del Litoral, ESPOL, Campus Gustavo Galindo Km 30.5 Vía Perimetral, Guayaquil, Ecuador; 5 National Graphene Institute, University of Manchester, Manchester, M13 9PL UK

Correction to: *Scientific Reports* 10.1038/s41598-023-36800-2, published online 26 June 2023

The original version of this Article contained an error in Figure 1 and 2. In Figure 1 the revised figure was not used in the article and in Figure 2b there was a typo in the y axis legend where “n × 10^–12^ (cm^-2^)” was changed to “n × 10^12^ (cm^-2^)”.

The original Figures 1 and 2 accompanying legends appear below.

**Figure 1:**
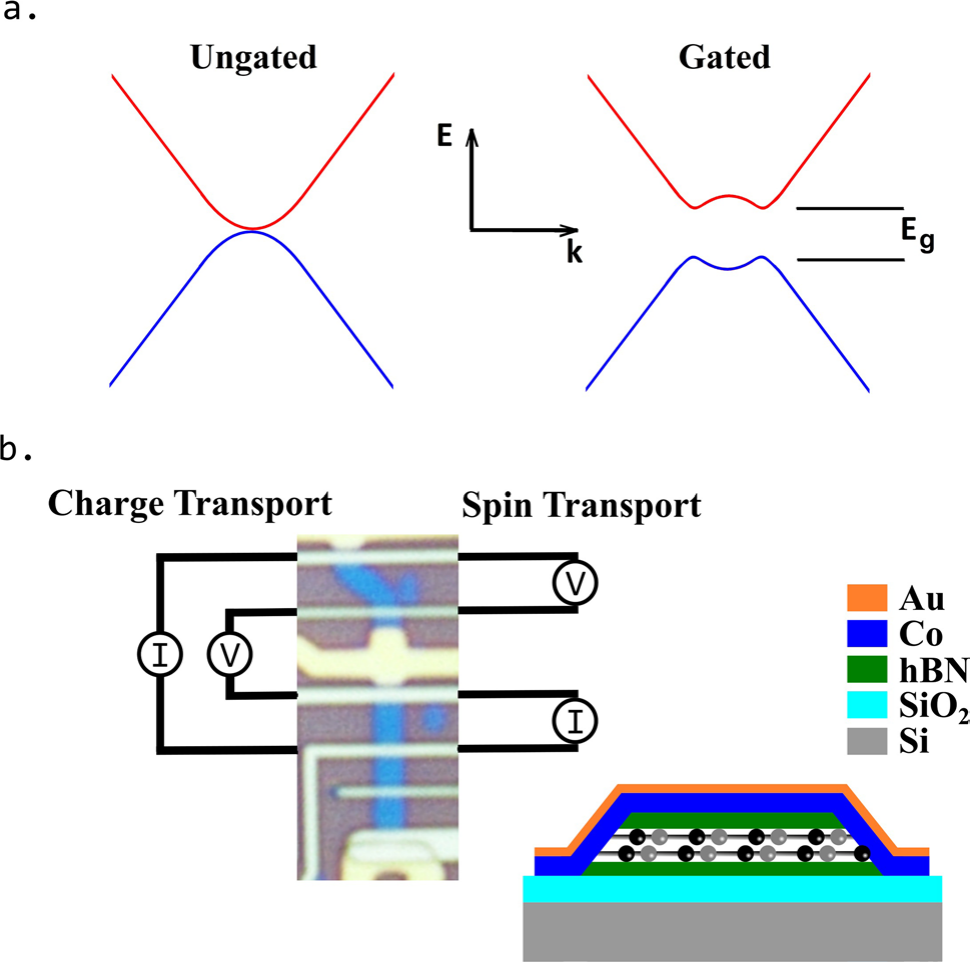
Bilayer graphene transport channel and device. (**a**) Band structure of pristine BLG without (left) and with (right) an applied perpendicular electric displacement field. (**b**) Optical micrograph of our ~ 1 µm wide BLG graphene transport channels (blue) with contacts and top gate (in the measurement region). The charge (spin) transport current injection, *I*, and local (non-local) potential difference, *V*, measurement configuration. Inset: Schematic of the device heterostructure showing the Co/Au 1D edge contacts, with the graphene represented by the balls and sticks.

**Figure 2:**
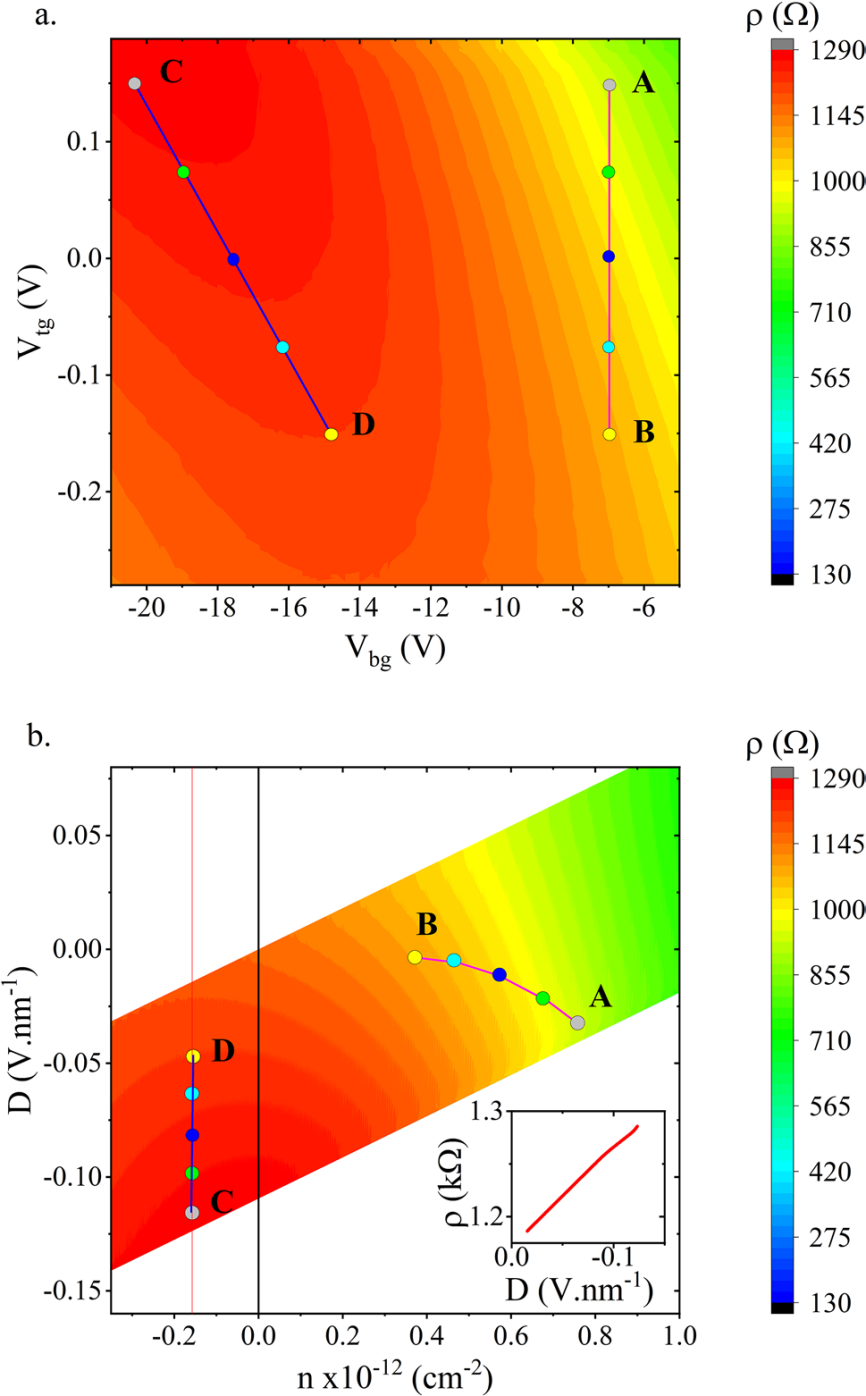
(**a**) A 2D charge transport measurement map at RT showing the effect on the sheet resistance, $\rho$, of applying a back gate voltage, $V_{bg}$, and top gate voltage, $V_{tg}$. (**b**) The same charge transport measurements transformed into a map as a function of carrier density, *n*, and electric displacement field, *D*. In both maps the symbols are shown where the spin transport measurements were made. b**(inset)** Sheet resistance close and parallel to the Dirac ridge ($n \approx 0$).

In addition, in the Results section, under the subheading ‘Spin transport measurements’,

“These are measurements where we sweep an in-plane magnetic field, which reverses the magnetisation of the 1D contacts and enables us to obtain either a parallel or antiparallel magnetic alignment between the injector and detector contacts.”,

now reads

“These are measurements where we sweep an in-plane magnetic field, B_‖_, applied along the direction of contacts, which reverses the magnetisation of the 1D contacts and enables us to obtain either a parallel or antiparallel magnetic alignment between the injector and detector contacts.”

Also,

“In doing so, we sweep a perpendicular magnetic field strength across a range of ±200 mT, which causes the diffusing electronic spins to experience Larmor precession, and fit the spin signal with the Hanle equation^22^.”

now reads

“In doing so, we sweep magnetic field strength perpendicular to the plane of the graphene (see Figure 1b), B_⊥_, across a range of ±200 mT, which causes the diffusing electronic spins to experience Larmor precession, and fit the spin signal with the Hanle equation^22^.”

The original Article has been corrected.

